# Analysis of Metabolic Risk Factors for Microcirculation Disorders Post-Percutaneous Coronary Intervention and Predictive Model Construction: A Study on Patients with Unstable Angina

**DOI:** 10.31083/RCM25739

**Published:** 2025-01-08

**Authors:** Kangming Li, Shuang Liu, Jing Wang, Zhen Liu, Chunmei Qi

**Affiliations:** ^1^Department of Cardiology, The Second Affiliated Hospital of Xuzhou Medical University, 221000 Xuzhou, Jiangsu, China

**Keywords:** coronary microvascular dysfunction, angiographic microvascular resistance, metabolic, unstable angina

## Abstract

**Background:**

This study aimed to analyze the metabolic risk factors for microcirculation disorders in patients with unstable angina (UA) after percutaneous coronary intervention (PCI), evaluating their predictive value for developing microcirculation disorders.

**Methods:**

A single-center retrospective study design was used, which included 553 patients with UA who underwent PCI. The angiographic microcirculatory resistance (AMR) index was calculated based on coronary angiography data. Patients were divided into two groups according to their post-PCI AMR values: a post-PCI AMR ≤2.50 group and a post-PCI AMR >2.50 group. Variables were included in the multivariate regression model through univariate regression and variance inflation factor (VIF) screening. Subgroup analyses were conducted by sex to further evaluate the predictive value of selected variables in the overall sample. The total sample was randomly split into a 7:3 ratio for the training and validation sets. A nomogram based on the training sets was then constructed to visualize these predictions. The discrimination and calibration of the prediction model were evaluated using the receiver operating characteristic (ROC) curve and calibration curve.

**Results:**

The post-PCI AMR >2.50 group had a higher percentage of females, increased incidence of diabetes, and elevated fasting blood glucose (FBG), glycated hemoglobin (HbA1c), triglyceride (TG), total cholesterol (TC), low-density lipoprotein cholesterol (LDL-C), very low-density lipoprotein cholesterol (VLDL-C), and lipoprotein(a) (Lp(a)) levels (*p* < 0.05). Logistic regression analysis identified HbA1c, TG, LDL-C, and Lp(a) as independent predictors of elevated AMR post-PCI after adjusting for confounders. Subgroup analysis confirmed no significant interaction between the model and sex (*p* > 0.05). A nomogram was constructed based on the training set, with the area under the curve (AUC) for the ROC of 0.824 in the training set and 0.746 in the validation set. The calibration curves showed a good fit (training set: *p* = 0.219; validation set: *p* = 0.258).

**Conclusions:**

HbA1c, TG, LDL-C, and Lp(a) levels are independent risk factors for microcirculation disorders in patients with UA post-PCI. The constructed nomogram provides good predictive accuracy.

## 1. Introduction

Acute coronary syndrome (ACS) is a major global public health issue with a high mortality rate. Unstable angina (UA), a critical component of ACS, represents a severe phenotype of coronary artery disease (CAD) [1]. UA presents as chest pain caused by an imbalance between myocardial oxygen supply and demand. Moreover, UA is a transitional state between stable angina and acute myocardial infarction. Notably, the clinical prognosis of patients with UA has significantly improved due to the continuous advancement of interventional techniques such as percutaneous coronary intervention (PCI) [2]. However, some patients still face the challenge of residual coronary ischemia after PCI, which further increases the risk of major adverse cardiovascular events [3, 4]. The index of microcirculatory resistance (IMR) is widely recognized in clinical practice as the “gold standard” for assessing coronary microcirculatory dysfunction due to its accuracy and reproducibility [5]. However, the measurement method for IMR is complex, requiring pressure wires, vasodilators, and repeated saline injections, extending the measurement time and increasing procedural risks. In recent years, angiographic microcirculatory resistance (AMR) has gained increasing attention as a potential alternative to IMR. Indeed, studies have shown that an AMR >2.5 can define coronary microcirculatory dysfunction and is closely associated with adverse cardiovascular events [6, 7, 8, 9]. AMR has been proven to quantify residual coronary ischemia as a parameter based on the quantitative flow fraction. Studies have also noted that an AMR >2.5 is associated with the occurrence of major adverse cardiovascular events post-procedure [6, 7]. Therefore, identifying and evaluating the metabolic factors that influence AMR after PCI is of significant clinical value for improving patient prognosis and reducing the risk of cardiovascular events.

With changes in modern lifestyle, the prevalence of metabolic disorders continues to rise. Metabolic diseases, particularly glucose and lipid metabolism disorders, have become global health challenges [10, 11]. Abnormalities in glucose and lipid metabolism not only increase the risk of cardiovascular diseases, such as coronary artery disease and heart failure but also contribute to dysfunction in various metabolic organs, constituting a complex systemic disorder [12, 13, 14]. Clinical biomarkers such as fasting blood glucose (FBG), glycated hemoglobin (HbA1c), and lipid profiles play crucial roles in diagnosing and screening glucose and lipid metabolism disorders. Further, these biomarkers provide important information for personalized treatment and drug development [15]. Although previous studies have shown an association between HbA1c, the triglyceride glucose (TyG) index, and coronary microcirculatory disorders [16, 17], these studies mostly rely on single metabolic indicators and lack a comprehensive evaluation of the overall correlation between glucose and lipid metabolism abnormalities and coronary microcirculatory disorders.

Given these limitations, this study aimed to analyze the association between glucose and lipid metabolism markers and coronary microcirculatory dysfunction in patients with UA after PCI. The goal was to identify high-risk patients following PCI and develop long-term management strategies to reduce the likelihood of future adverse cardiovascular events.

## 2. Materials and Methods

### 2.1 Study Population

This study included patients with UA who were admitted to the Department of Cardiology, Second Affiliated Hospital of Xuzhou Medical University, between June 2022 and February 2024 and underwent successful PCI.

The inclusion criteria were as follows: (1) admission due to UA and underwent PCI; (2) preoperative use of aspirin, ticagrelor, and statins.

The exclusion criteria were as follows: (1) age <18 years; (2) history of myocardial infarction or cardiomyopathy; (3) history of coronary artery bypass grafting; (4) thyroid dysfunction (hypothyroidism or hyperthyroidism); (5) liver cirrhosis, anemia, or dialysis; (6) incomplete baseline data.

A total of 654 patients with UA were initially assessed, and after applying the exclusion criteria, 553 patients were included in this study. This single-center, retrospective study was conducted in accordance with the Helsinki Declaration and approved by the Ethics Committee of the Second Affiliated Hospital of Xuzhou Medical University (2020120205). The patients and their families were informed about the study, and all participants provided written informed consent.

### 2.2 Data Collection

**Fig. 1.**
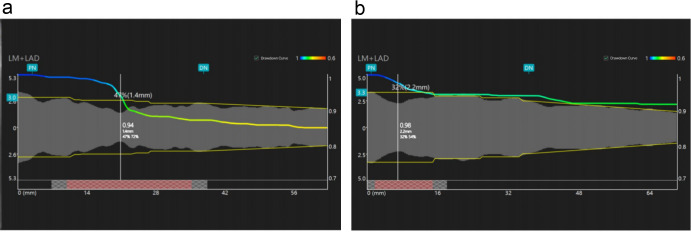
**Angiographic microcirculatory resistance (AMR) measurements in post-PCI patients**. (a) Post-percutaneous coronary intervention (PCI) patient AMR = 2.65 mmHg*s/cm. (b) Post-PCI patient AMR = 1.98 mmHg*s/cm. LM, left main coronary artery; LAD, left anterior descending branch; PN, proximal node; DN, distal node.

Patient demographics included age, sex, and medical history (smoking, alcohol consumption, history of prior percutaneous coronary intervention, hypertension, diabetes mellitus); laboratory tests included peripheral blood white blood cells (WBC), hemoglobin (Hb), FBG, HbA1c, total cholesterol (TC), triglycerides (TGs), high-density lipoprotein cholesterol (HDL-C), low-density lipoprotein cholesterol (LDL-C), very low-density lipoprotein cholesterol (VLDL-C), apolipoprotein A1 (Apo A1), apolipoprotein B (Apo B), apolipoprotein E (Apo E), lipoprotein(a) (Lp(a)), homocysteine (HCY); echocardiography parameters included left atrial anteroposterior diameter (LAAPD), left ventricular anteroposterior diameter (LVAPD), interventricular septum thickness (IVS), left ventricular posterior wall thickness (LVPW), right ventricular anteroposterior diameter (RVAPD), left ventricular ejection fraction (LVEF). The post-PCI AMR values for patients with UA were calculated and classified into two groups: post-PCI AMR ≤2.50 (Fig. 1a) and post-PCI AMR >2.50 (Fig. 1b).

AMR was calculated using quantitative flow ratio (QFR) software (AngioPlus Core, version V3, Shanghai Pulse Medical Technology Inc., Shanghai, China) through a single-view Murray’s law-based QFR (µQFR) analysis. The single-view µQFR calculation method involved using QFR software to delineate the contours of the coronary artery lumen. The single-view µQFR calculation method was performed by dividing the length of the vessel centerline by the contrast agent filling time to compute the hyperemic flow velocity [18]. Subsequently, fully contrasted and exposed lumen contour frames were selected to outline the boundaries of the vessels and the major side branches of the examined patient. The reference vessel diameter was reconstructed by considering the phenomenon of diameter reduction at bifurcations based on Murray’s branching fractal law [19]. Finally, using hyperemic flow as a boundary condition, the pressure drop was calculated based on the fluid dynamics equation. The distal coronary pressure (Pd) was obtained based on the pressure drop, and the µQFR was calculated as Pd divided by the mean aortic pressure (Pa). The AMR was calculated as Pd divided by the simulated maximum flow velocity under hyperemic conditions (hyperemic flow velocity, V_hyp_) [20].

AMR = Pd/V_hyp_ = Pa × µQFR/V_hyp_

### 2.3 Data Analysis

SPSS 27.0 (IBM Corp., Armonk, NY, USA) and R 4.3.1 (The R Foundation for Statistical Computing, Vienna, Austria) statistical analysis software were used. Normally distributed data were assessed using the Shapiro–Wilk test and are presented as the mean ± standard deviation ($\bar{x}$ ± s). Non-normally distributed data were analyzed using the Wilcoxon test and are presented as the median (M) and interquartile range (IQR). Categorical variables were analyzed using the chi-squared test and are presented as percentages (%). Single-factor regression analysis was used to screen for differences in various indicators affecting post-PCI AMR. Indicators with *p* < 0.05 were included in a multiple regression model to analyze the independent association between glucose and lipid metabolism biomarkers and post-PCI AMR.

## 3. Results

### 3.1 Comparison of Clinical Data Between Two Groups

This study included 553 patients with UA. Compared with patients presenting a post-PCI AMR ≤2.50, the proportion of females and those with diabetes was higher in patients with a post-PCI AMR >2.5 (*p* < 0.05). The FBG, HbA1c, TG, TC, LDL-C, VLDL-C, and Lp(a) levels were also higher in the latter group (Table 1).

**Table 1.** **Comparison of baseline data between two groups of patients**.

Variables		Post-PCI	Post-PCI	*p*
		AMR ≤2.50	AMR >2.50	
		(n = 279)	(n = 274)	
Age, years		68.5 (60–74)	68 (57–73)	0.197
Female		89 (43.8%)	114 (56.2%)	0.041
Medical history				
	Smoking	91 (51.1%)	87 (48.9%)	0.610
	Alcohol use	58 (50.4%)	57 (49.6%)	0.831
	Hypertension	179 (50.7%)	174 (49.3%)	0.468
	DM	64 (34.2%)	123 (65.8%)	<0.001
	History of PCI	98 (54.1%)	83 (45.9%)	0.132
Laboratory tests				
	WBC, 10^9^/L	6.38 (5.2–7.67)	6.33 (5.24–7.85)	0.480
	Hb, g/L	135 (124–145)	136 (126–149)	0.146
	FBG, mmol/L	5.24 (4.69–5.86)	6.12 (5.05–7.25)	<0.001
	HbA1c, g/L	5.9 (5.5–6.3)	6.4 (5.7–7.4)	<0.001
	Creatinine, µmol/L	68 (60–82)	67 (58–79)	0.316
	TG, mmol/L	1.23 (0.89–1.66)	1.51 (1.11–2.19)	<0.001
	TC, mmol/L	3.99 ± 1.03	4.75 ± 1.32	<0.001
	HDL-C, mmol/L	1.08 (0.94–1.26)	1.09 (0.93–1.32)	0.530
	LDL-C, mmol/L	1.79 (1.44–2.32)	2.48 (1.8–3.04)	<0.001
	VLDL-C, mmol/L	0.65 (0.44–0.93)	0.71 (0.52–1.09)	0.013
	Apo A1, g/L	1.24 (1.05–1.40)	1.24 (1.08–1.45)	0.479
	Apo B, g/L	1.25 ± 0.32	1.58 ± 0.30	0.452
	Apo E, mg/L	37.5 (29.88–48)	40 (31–50)	0.109
	Lp(a), mg/dL	14.25 (6.2–24.73)	22.5 (9.7–46.2)	<0.001
	HCY, µmol/L	14.7 (11.95–18.85)	14.4 (12–18.5)	0.847
Myocardial enzyme				
	CTnI, ng/mL	0.004 (0.002–0.0101)	0.004 (0.002–0.0082)	0.672
	CK, U/L	67 (48–93)	66 (46–93)	0.957
	CK-MB, ng/mL	0.8 (0.6–1.3)	0.8 (0.6–1.2)	0.370
Echocardiography				
	LAAPD, mm	36.38 ± 6.93	35.92 ± 7.83	0.460
	LVAPD, mm	46 (43–48)	46 (42–48)	0.868
	IVS, mm	9.75 (9–10)	9 (9–10)	0.507
	LVPW, mm	9 (9–10)	9 (8–10)	0.268
	RVAPD, mm	22 (21–24)	22 (21–24)	0.428
	LVEF, %	59 (57–60)	59 (57–60)	0.240

DM, diabetes mellitus; WBC, white blood cells; HbA1c, glycated hemoglobin; FBG, fasting blood glucose; TG, triglyceride; TC, total cholesterol; HDL-C, high-density lipoprotein cholesterol; LDL-C, low-density lipoprotein cholesterol; VLDL-C, very low-density lipoprotein cholesterol; Apo A1, apolipoprotein A1; Apo B, apolipoprotein B; Apo E, apolipoprotein E; Lp(a), lipoprotein(a); HCY, homocysteine; LAAPD, left atrial anteroposterior diameter; LVAPD, left ventricular anteroposterior diameter; IVS, interventricular septum thickness; LVPW, left ventricular posterior wall thickness; RVAPD, right ventricular anteroposterior diameter; LVEF, left ventricular ejection fraction; Hb, hemoglobin; PCI, percutaneous coronary intervention; AMR, angiographic microcirculatory resistance; CTnI, cardiac troponin I; CK, creatine kinase; CK-MB, creatine kinase isoenzyme MB.

### 3.2 Factor Analysis

The results of single-factor logistic regression analysis of factors influencing AMR indicated that female sex (OR = 1.436, 95% CI: 1.014–2.033, *p* = 0.041) and diabetes mellitus (OR = 2.587, 95% CI: 1.794–3.732, *p* < 0.001), and higher FBG (OR = 1.526, 95% CI: 1.336–1.744, *p* < 0.001), HbA1c (OR = 1.982, 95% CI: 1.630–2.410, *p* < 0.001), TG (OR = 1.588, 95% CI: 1.291–1.954, *p* < 0.001), TC (OR = 1.812, 95% CI: 1.530–2.147, *p* < 0.001), LDL-C (OR = 2.457, 95% CI: 1.943–3.105, *p* < 0.001), VLDL-C (OR = 1.504, 95% CI: 1.055–2.144, *p* = 0.024), and Lp(a) levels (OR = 1.017, 95% CI: 1.010–1.025, *p* < 0.001) were significantly associated with an increased risk of AMR >2.5 post-PCI (*p* < 0.05) (Table 2).

**Table 2.** **Univariate analysis of risk factors for AMR >2.5 after PCI in patients with UA**.

Variables	OR	95% CI	*p*
Age, years	0.989	1.975–1.004	0.154
Female	1.436	1.014–2.033	0.041
Smoking	1.097	0.768–1.568	0.610
Alcohol use	1.064	0.705–1.608	0.766
Hypertension	1.137	0.803–1.609	0.469
DM	2.587	1.794–3.732	<0.001
History of PCI	0.761	0.533–1.086	0.132
WBC, 10^9^/L	1.046	0.974–1.125	0.217
Hb, g/L	1.009	0.999–1.019	0.086
FBG, mmol/L	1.526	1.336–1.743	<0.001
HbA1c, g/L	1.982	1.630–2.410	<0.001
Creatinine, µmol/L	1.000	0.997–1.002	0.743
TG, mmol/L	1.588	1.291–1.954	<0.001
TC, mmol/L	1.812	1.530–2.147	<0.001
HDL-C, mmol/L	1.285	0.774–2.134	0.332
LDL-C, mmol/L	2.457	1.943–3.105	<0.001
VLDL-C, mmol/L	1.504	1.055–2.144	0.024
Apo A1, g/L	1.330	0.713–2.483	0.370
Apo B, g/L	1.013	0.979–1.048	0.460
Apo E, mg/L	1.008	1.000–1.016	0.058
Lp(a), mg/dL	1.017	1.010–1.025	<0.001
HCY, µmol/L	1.005	0.990–1.020	0.533
LAAPD, mm	0.991	0.969–1.014	0.459
LVAPD, mm	1.008	0.972–1.045	0.672
IVS, mm	0.951	0.844–1.071	0.408
LVPW, mm	0.914	0.787–1.062	0.240
RVAPD, mm	0.966	0.900–1.037	0.340
LVEF, %	1.014	0.979–1.049	0.439

UA, unstable angina; DM, diabetes mellitus; WBC, white blood cells; HbA1c, glycated hemoglobin; FBG, fasting blood glucose; TG, triglyceride; TC, total cholesterol; HDL-C, high-density lipoprotein cholesterol; LDL-C, low-density lipoprotein cholesterol; VLDL-C, very low-density lipoprotein cholesterol; Apo A1, apolipoprotein A1; Apo B, apolipoprotein B; Apo E, apolipoprotein E; Lp(a), lipoprotein(a); HCY, homocysteine; LAAPD, left atrial anteroposterior diameter; LVAPD, left ventricular anteroposterior diameter; IVS, interventricular septum thickness; LVPW, left ventricular posterior wall thickness; RVAPD, right ventricular anteroposterior diameter; LVEF, left ventricular ejection fraction; Hb, hemoglobin; PCI, percutaneous coronary intervention; AMR, angiographic microcirculatory resistance.

**Fig. 2.**
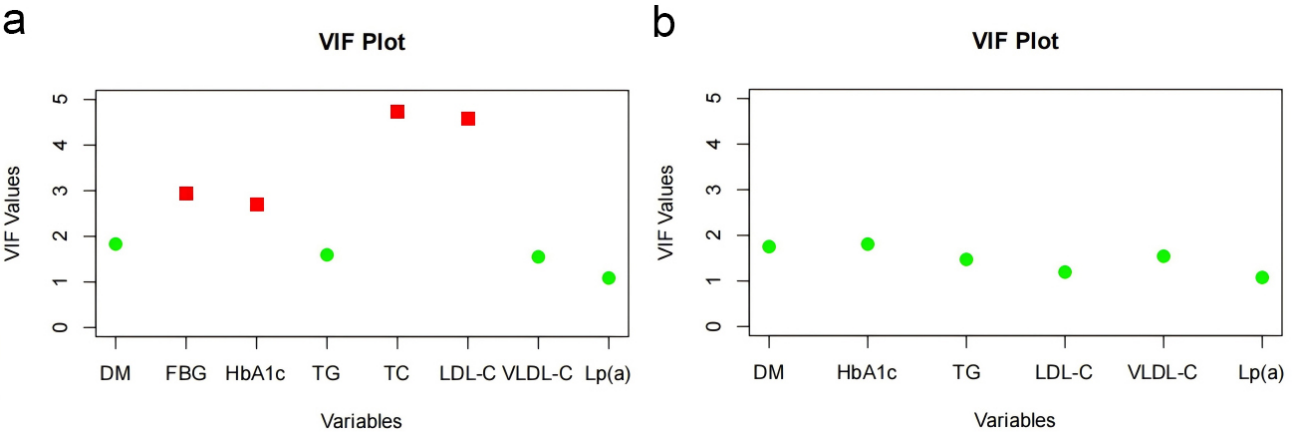
**Variance inflation factor (VIF) comparison before and after variable adjustment**. (a) VIF chart before variable adjustment. (b) VIF chart after variable adjustment. DM, diabetes mellitus; HbA1c, glycated hemoglobin; FBG, fasting blood glucose; TG, triglyceride; TC, total cholesterol; LDL-C, low-density lipoprotein cholesterol; VLDL-C, very low-density lipoprotein cholesterol; Lp(a), lipoprotein(a).

Before including variables with a value of *p* < 0.05 from the univariate analysis into the multivariate logistic regression, we conducted a collinearity analysis on DM, FBG, HbA1c, TG, TC, LDL-C, VLDL-C, and Lp(a) levels. Collinearity was found between FBG and HbA1c (variance inflation factor (VIF) >2), as well as TC and LDL-C (VIF >2). By removing the variables with high VIF values, we reduced the VIF of the model to an acceptable level (Fig. 2).

**Fig. 3.**
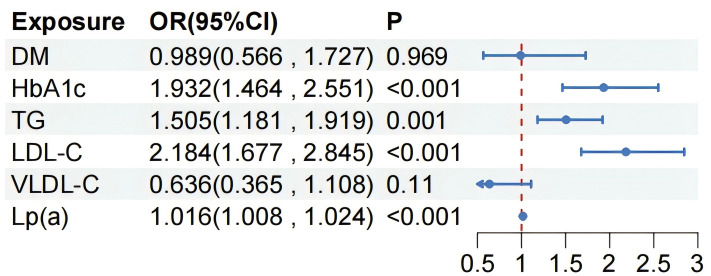
**Multivariate analysis of risk factors with an AMR >2.5 in patients with UA after PCI**. UA, unstable angina; PCI, percutaneous coronary intervention; AMR, angiographic microcirculatory resistance; DM, diabetes mellitus; HbA1c, glycated hemoglobin; TG, triglyceride; LDL-C, low-density lipoprotein cholesterol; VLDL-C, very low-density lipoprotein cholesterol; Lp(a), lipoprotein(a).

We included variables with a value of *p* < 0.05 from the univariate analysis in the multivariate logistic regression (Fig. 3) and found that HbA1c (OR = 1.932, 95% CI: 1.464–2.551, *p* < 0.001), TG (OR = 1.505, 95% CI: 1.181–1.919, *p* = 0.001), LDL-C (OR = 2.184, 95% CI: 1.677–2.845, *p* < 0.001), and Lp(a) levels (OR = 1.016, 95% CI: 1.008–1.024, *p* < 0.001) were independent risk factors for post-PCI AMR >2.5.

**Fig. 4.**
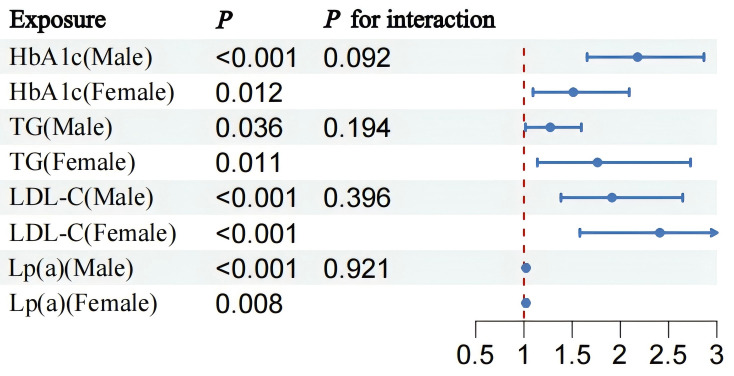
**Statistical comparison of HbA1c, TG, LDL-C, and Lp(a) levels as indicators in males and females**. HbA1c, glycated hemoglobin; TG, triglyceride; LDL-C, low-density lipoprotein cholesterol; Lp(a), lipoprotein(a).

Subgroup analysis confirmed that there was no significant interaction between the model and sex (*p* > 0.05), indicating the stability of the model was not influenced by sex (Fig. 4).

**Fig. 5.**
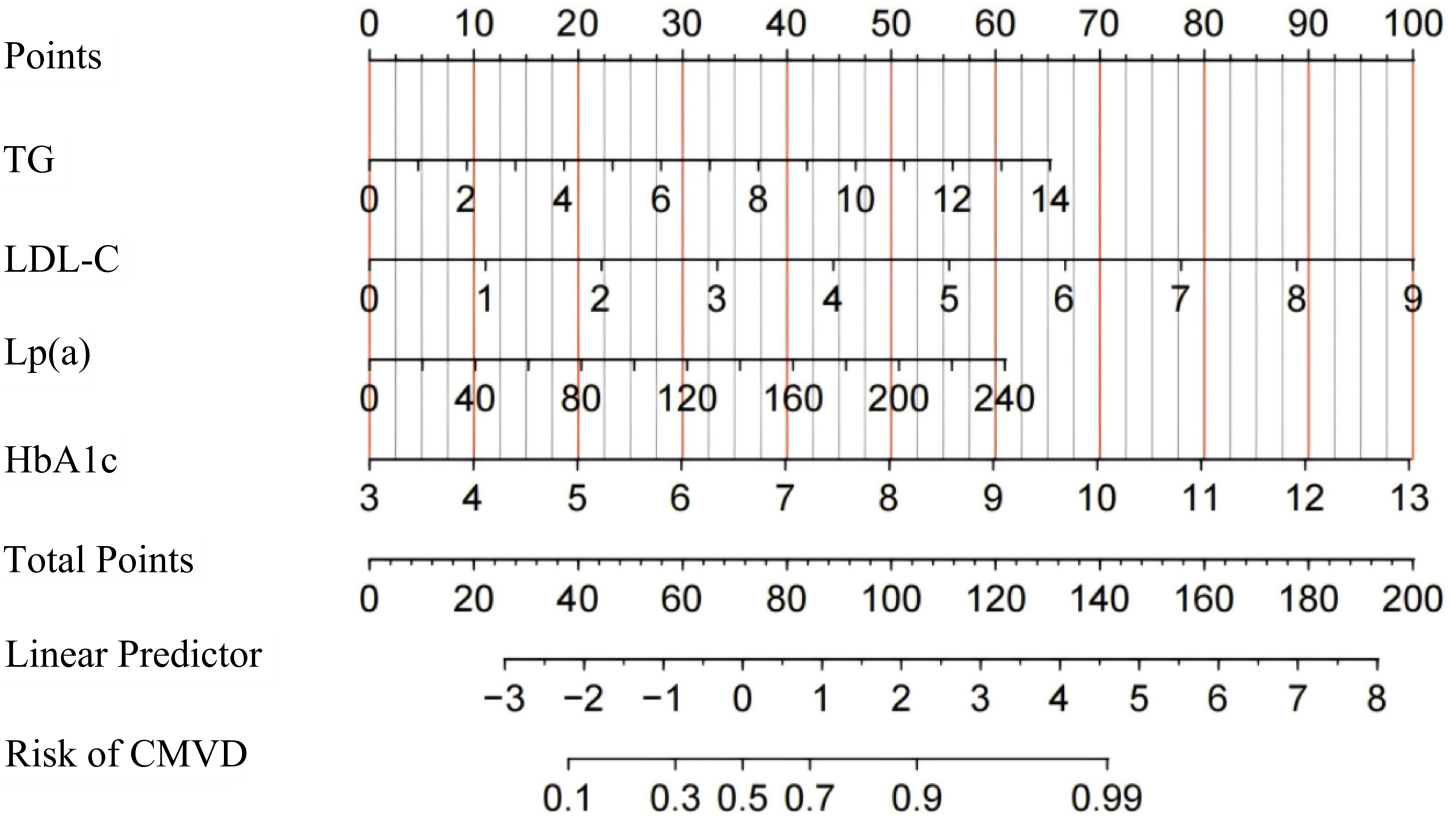
**Nomogram for diagnosing coronary microvascular dysfunction after PCI in patients with UA**. UA, unstable angina; PCI, percutaneous coronary intervention; CMVD, coronary microvascular disease; HbA1c, glycated hemoglobin; TG, triglyceride; LDL-C, low-density lipoprotein cholesterol; Lp(a), lipoprotein(a).

The samples were randomly divided into training and validation sets in a 7:3 ratio. Four independent risk factors were used to create a nomogram based on a training set of logistic regression coefficients to construct a risk estimate of coronary microcirculatory disturbance in patients with UA after PCI (Fig. 5). As shown in Fig. 2, the selected biomarkers were scored using the values obtained from the predictive model. Fig. 2 illustrates the predictions of coronary microvascular dysfunction after PCI in patients with UA at different biomarker levels using a linear prediction model and their corresponding risk levels. As the biomarker scores increased, the predicted risk values also significantly increased, suggesting a close association between these biomarkers and the risk of coronary microvascular dysfunction after PCI in patients with UA.

**Fig. 6.**
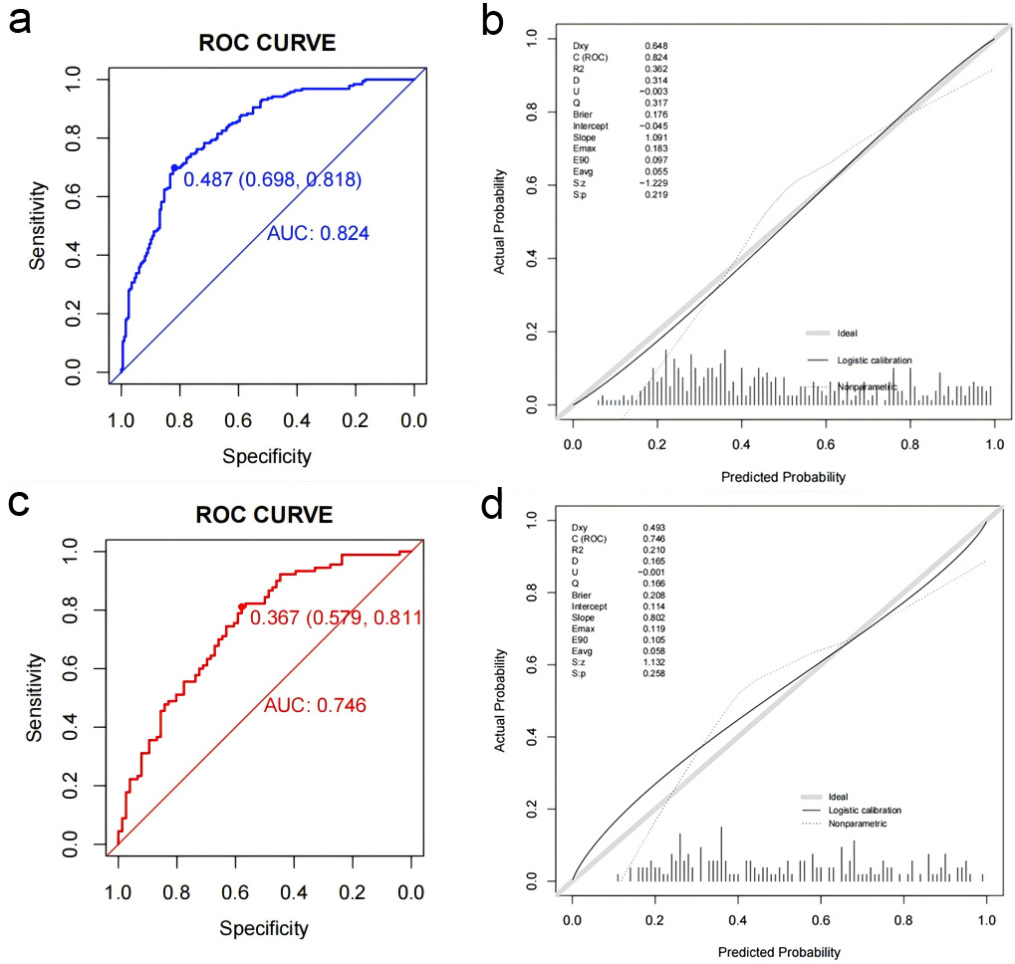
**Receiver operating characteristic curves and calibration plots of the nomogram in the training and validation set**. (a) ROC curve for predicting coronary microcirculation disorder post-PCI in the training set. (b) Calibration curve for the training set. (c) ROC curve for predicting coronary microcirculation disorder post-PCI in the validation set. (d) Calibration curve for the validation set. PCI, percutaneous coronary intervention; ROC, receiver operating characteristic; AUC, area under the curve.

The discrimination ability of the nomogram model was assessed using the receiver operating characteristic (ROC) curve. In the training set, the model achieved an area under the curve (AUC) for the ROC of 0.824 with an optimal cutoff of 0.487, yielding a sensitivity of 0.818 and a specificity of 0.698. In the validation set, the AUC was 0.746, with an optimal cutoff of 0.367, sensitivity of 0.811, and specificity of 0.579 (Fig. 6a,c). Calibration of the model was assessed using the Hosmer–Lemeshow test, with results of *p* = 0.219 for the training set and *p* = 0.258 for the validation set (both *p* > 0.05), indicating good model consistency (Fig. 6b,d).

## 4. Discussion

UA, a disease that significantly affects patients’ quality of life, has long been a focus of cardiovascular treatment and management. Despite significant advancements in PCI technology improving patient outcomes, residual coronary ischemia remains a critical factor leading to adverse cardiovascular events [4, 21, 22]. Yongzhen Fan *et al*. [23] validated the effectiveness of AMR in diagnosing coronary microcirculatory dysfunction, highlighting an AMR >2.5 as a critical threshold for this disorder. Previous studies have demonstrated that AMR effectively assesses coronary artery physiological function and correlates closely with an increased risk of future adverse cardiovascular events [6, 7, 8, 9]. Therefore, identifying and evaluating metabolic susceptibility factors affecting post-PCI AMR and determining high-risk patients after PCI can help develop personalized long-term management strategies to reduce the likelihood of adverse cardiovascular events in the future.

The root cause of glycemic and lipid metabolism disorders stems from the imbalance between pro-oxidant and antioxidant levels, leading to endothelial dysfunction promoting cardiovascular disease development [10]. Biological markers, as indicators for disease diagnosis and staging, allow the detection of changes in patient biomarkers and can develop personalized treatments [15]. Moreover, FBG, HbA1c, and lipid profiles are biological markers of glycemic and lipid metabolism and are significantly associated with the risks of cardiovascular events [13, 14, 22, 24]. HbA1c is the gold standard for long-term blood glucose control, reflecting the glycemic status over the past 2–3 months. Prolonged hyperglycemia results in advanced glycation end products (AGEs) that inhibit endothelial nitric oxide synthase (eNOS) and induce vascular and myocardial collagen cross-linking, thereby impairing vascular dilation [25]. The study have confirmed a significant correlation between HbA1c levels and residual coronary ischemia post-PCI [17]. Furthermore, research indicates that optimal control of LDL-C in patients undergoing PCI can significantly improve coronary microvascular function after one year due to a reduction in LDL-C, which improves endothelial cell function [26, 27]. Most studies analyze the correlation between a single metabolic abnormality and cardiovascular events, meaning the close relationship between glycemic and lipid metabolism abnormalities is often overlooked in single-angle analyses [28, 29]. Therefore, analyzing the independent risk factors for residual coronary ischemia post-PCI in patients with UA from a comprehensive glycemic and lipid metabolism perspective and conducting subgroup analysis can provide more precise clinical guidance. This comprehensive analytical approach helps to improve understanding of the overall impact of glycemic and lipid metabolism abnormalities on cardiovascular events, enabling the development of more effective personalized treatment strategies.

This study thoroughly explored the relationship between glucose and lipid metabolism disorders and microcirculatory dysfunction in patients with UA after PCI and evaluated the predictive value of relevant metabolic indicators. The results revealed that HbA1c, TG, LDL-C, and Lp(a) levels can be independent risk factors for post-PCI microcirculatory dysfunction. Subgroup analyses based on sex further demonstrated that these risk factors, identified in the overall sample, remained significant across both male and female groups. This indicates the robustness of these indicators in predicting post-PCI AMR >2.5, confirming their general applicability regardless of sex, with no significant interaction effects. To further evaluate the predictive value of these risk factors, the total sample was randomly divided into a training set and a validation set at a 7:3 ratio. A nomogram including HbA1c, TG, LDL-C, and Lp(a) was constructed based on the training set. The predictive performance of the nomogram was assessed using ROC curves, presenting an AUC of 0.824 in the training set, indicating high accuracy. The model was then tested in the validation set, with an AUC of 0.746, again demonstrating high accuracy. Therefore, calibration curves in both the training and validation sets confirmed the consistency of the model. Furthermore, these indicators are both accessible and affordable, allowing for risk assessment in patients with UA post-PCI and the development of individualized treatment plans and management strategies to reduce the likelihood of future cardiovascular adverse events.

The findings of this study have several important implications for clinical practice. First, they emphasize the importance of monitoring and controlling metabolic risk factors in managing patients with UA post-PCI. Second, the predictive model based on the nomogram helps in the early identification of high-risk patients and facilitates timely interventions.

### Limitations

The results of this study also have some limitations. First, our model is based on a clinical single-institution study with only 553 patients with UA post-PCI, meaning the findings may not be generalizable to the broader population. Second, while previous studies have confirmed that an AMR score >2.5 can define coronary microcirculatory dysfunction compared with IMR, large-scale clinical studies remain lacking in validating this concept. Third, AMR measurements may be inaccurate in cases of severe coronary artery stenosis and when there is a lack of preoperative assessment of the microcirculatory function of patients.

## 5. Conclusions

HbA1c, TG, LDL-C, and Lp(a) are independent risk factors for microcirculatory dysfunction after PCI in patients with UA. A predictive model incorporating these factors was established, demonstrating good predictive value and clinical utility, which can provide clinicians with valuable information to develop treatment strategies to minimize future major adverse cardiovascular events.

## Data Availability

The datasets used and/or analyzed during the current study are available from the corresponding author on reasonable request.

## Abbreviations

UA, unstable angina pectoris; PCI, percutaneous coronary intervention; QFR, quantitative flow ratio; IMR, index of microcirculatory resistance; AMR, angiographic microvascular resistance; CMVD, coronary microvascular disease; DM, diabetes mellitus; TG, triglyceride; HDLC, high-density lipoprotein cholesterol; LDLC, low-density lipoprotein cholesterol; VLDL, very low-density lipoprotein; FBG, fasting blood glucose; HbA1c, glycated hemoglobin.
